# Three missense variants of metabolic syndrome-related genes are associated with alpha-1 antitrypsin levels

**DOI:** 10.1038/ncomms8754

**Published:** 2015-07-15

**Authors:** Kazuya Setoh, Chikashi Terao, Shigeo Muro, Takahisa Kawaguchi, Yasuharu Tabara, Meiko Takahashi, Takeo Nakayama, Shinji Kosugi, Akihiro Sekine, Ryo Yamada, Michiaki Mishima, Fumihiko Matsuda

**Affiliations:** 1 Center for Genomic Medicine, Kyoto University Graduate School of Medicine, Shogoinkawaramachi 53, Sakyo-ku, Kyoto 606-8507, Japan.; 2 Department of Respiratory Medicine, Kyoto University Graduate School of Medicine, Shogoinkawaramachi 54, Sakyo-ku, Kyoto 606-8507, Japan.; 3 Department of Health Informatics, Kyoto University School of Public Health, Yoshidakonoemachi, Sakyo-ku, Kyoto 606-8501, Japan.; 4 Department of Medical Ethics and Medical Genetics, Kyoto University School of Public Health, Yoshidakonoemachi, Sakyo-ku Kyoto, 606-8501, Japan.; 5 EBM Research Center, Kyoto University Graduate School of Medicine, Shogoinkawaramachi 54, Sakyo-ku, Kyoto 606-8507, Japan.; 6 Statistical Genetics, Center for Genomic Medicine, Kyoto University Graduate School of Medicine, Shogoinkawaramachi 53, Sakyo-ku, Kyoto 606-8507, Japan.

## Abstract

Alpha-1 antitrypsin (AAT) encoded by *SERPINA1* is an acute-phase inflammation marker, and AAT deficiency (AATD) is known as one of the common genetic disorders in European populations. However, no genetic determinants to AAT levels apart from the *SERPINA* gene clusters have been identified to date. Here we perform a genome-wide association study of serum AAT levels followed by a two-staged replication study recruiting a total of 9,359 Japanese community-dwelling population. Three missense variants of metabolic syndrome-related genes, namely, rs671 in *ALDH2*, rs1169288 in *HNF1A* and rs1260326 in *GCKR*, significantly associate with AAT levels (*P*≤1.5 × 10^−12^). Previous reports have shown the functional relevance of *ALDH2* and *HNF1A* to AAT. We observe a significant interaction of rs671 and alcohol consumption on AAT levels. We confirm the association between AAT and rs2896268 in *SERPINA1*, which is independent of known causative variants of AATD. These findings would support various AAT functions including metabolic processes.

## Abstract (web summary)

Alpha-1 antitrypsin deficiency is a common genetic disorder in Europeans. Here Setoh *et al*. perform a genome-wide association study of AAT serum levels in a Japanese population and find three missense variants in the metabolism genes *ALDH2*, *HNF1A* and *GCKR*.

## Graphical abstract

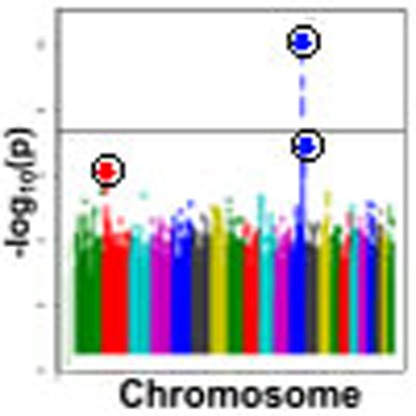

Alpha-1 antitrypsin (AAT) is an acute-phase inflammation marker synthesized predominantly in hepatocytes and serves as a protease inhibitor of proteolytic enzymes including elastase, trypsin, thrombin and bacterial proteases^1^,^2^. Recent studies have revealed the wide range of functions for AAT^3^,^4^,^5^. AAT is encoded by the *SERPINA1* gene on chromosome 14 (ref. 6). The reduction of AAT levels leads to inflammation in the lung, and is associated with the risk for early-onset chronic obstructive pulmonary disease (COPD)^7^,^8^,^9^. AAT deficiency (AATD) is a common genetic disorder among Caucasian populations caused by mutations in *SERPINA1*. Patients with AATD present with emphysema due to imbalance of elastase and anti-elastase effect of AAT, and, in some cases, accumulating AAT affects hepatic functions and leads to liver cirrhosis and hepatocellular carcinoma^10^,^11^. Many variants in *SERPINA1* were reported to cause AATD^12^. The S variant (PI S, rs17580) and the Z variant (PI Z, rs28929474) are the major missense single-nucleotide polymorphisms (SNPs) leading to AATD by moderate and severe decline of AAT levels, respectively, through the conformational change of the AAT protein and the subsequent faulty secretion of AAT from hepatocytes^12^. All other variants reported to cause AATD are rare variants in *SERPINA1* (ref. 12).

Genome-wide association studies (GWAS) have enabled us to detect susceptibility loci associated with quantitative traits as well as discrete phenotypes using an unbiased approach. To date, only one GWAS has been reported in an European population to detect susceptibility loci to serum AAT levels, and the authors identified markers in the *SERPINA* gene cluster on 14q32.13 (ref. 13). Thus, there are no other markers reported to be associated with serum AAT levels. Novel susceptibility loci would uncover mechanisms of AAT metabolism and molecular networks for regulation of inflammatory processes in the lung.

As genetic backgrounds differ between different populations, GWAS using populations other than European populations would lead to identify novel susceptibility loci to serum AAT levels. Here we perform GWAS and a two-staged replication study of serum AAT levels using 9,359 Japanese healthy individuals participating in the Nagahama Study and show a total of three missense variants, rs671, rs1169288 and rs1260326 in metabolic syndrome-related genes, *ALDH2*, *HNF1A* and *GCKR*, respectively. We also show a significant interaction of rs671 and alcohol consumption on AAT levels. In addition, we show rs2896268 in *SERPINA1* as an AAT-associated variant independent of known causative variants of AATD.

## Results

### GWAS of serum AAT levels

**Figure 1:**
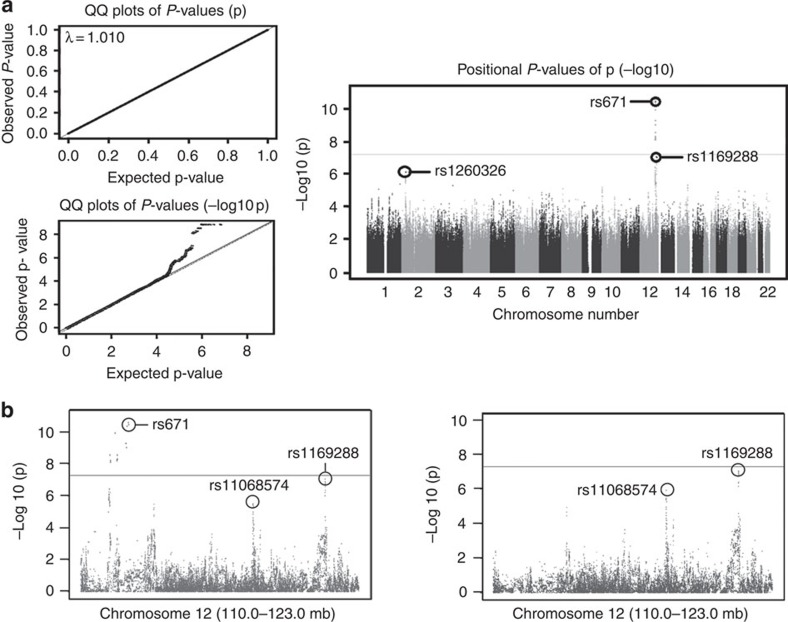
The results of GWAS on the serum AAT levels (*n*=3,294). (**a**) The quantile-quantile plots (left) and the Manhattan plot for the genome-wide scanning: The horizontal line indicates the genome-wide significant level, *P*=5.0 × 10^−8^. (**b**) The regional plot for a part of chromosome 12 for nominal (left) and conditional (right) associations adjusted for rs671 genotypes. *P* values were calculated by generalized linear regression model.

We conducted genome scanning using 3,693 participants of the Nagahama Study, a prospective Japanese cohort of community-dwelling population^14^, with the use of Illumina Infinium arrays to identify genetic loci affecting serum AAT levels in the Japanese population. The serum AAT levels did not show a deviated distribution (Supplementary Fig. 1). We used generalized linear regression model (GLM) with covariates including age, sex, body-mass index (BMI), C-reative protein (CRP), the recruiting years and smoking status (for details, see Methods). We performed genotype imputation by using the East Asian panel in the 1000 Genome Project^15^ as a reference. After quality control, 3,294 subjects and 6,569,727 SNPs were used for the association study (Table 1, for details, see Methods and Supplementary Figs 2 and 3). The quantile-quantile plots of the results of the genome scanning revealed no population structure in the current study (genomic inflation factor *λ*=1.01, Fig. 1a). Thus, we did not apply genomic control^16^ into the association study. As a result, rs671 in *ALDH2* on chromosome 12 showed a significant association beyond the GWAS significant level (GLM *P*=3.4 × 10^−11^, Fig. 1a and Table 2). Three additional loci, namely, the *HNF1A* and *KSR2* regions on chromosome 12 and the *GCKR* region on chromosome 2 showed suggestive associations with *P* values less than 1.0 × 10^−6^ (GLM *P*=8.5 × 10^−8^, 9.8 × 10^−7^ and 7.1 × 10^−7^, respectively, Fig. 1a and Table 2). All SNPs showing *P* values less than 1.0 × 10^−6^ are shown in Supplementary Table 1. rs1169288 on *HNF1A* and rs11068574 on *KSR2* were 9.2 and 5.8 Mbp apart from rs671, respectively, and not in linkage disequilibrium (LD) with rs671 (*r*^2^≤0.002, Supplementary Fig. 4). We confirmed that these two associations were independent from rs671 by conditioning on rs671 (GLM *P*<2.0 × 10^−6^, Fig. 1b). When we conditioned the associations in the four regions by each SNP (rs671, rs1169288, rs11068574 and rs1260326), no further association signals remained (GLM *P*≥0.0072, Supplementary Fig. 5). Thus, we selected these four markers to be genotyped in the replication studies as candidate markers affecting serum AAT levels.

### Two replication studies and combined study

We conducted a replication study to genotype these four SNPs using 4,023 subjects in the Nagahama Study by Taqman assay (Table 1 and Supplementary Fig. 2). As a result, the associations between serum AAT levels and rs671, rs1169288 or rs1260326 were replicated with *P* values less than 2.7 × 10^−4^ (GLM *P*=2.2 × 10^−10^, 2.7 × 10^−4^ and 2.3 × 10^−7^, respectively, Table 2). The association between rs11068574 and serum AAT levels was not replicated (GLM *P*=0.22, Table 2).

We further performed a second replication for these SNPs using 2,042 subjects by Taqman assay (Table 1 and Supplementary Fig. 2). As a result, the associations of rs671, rs1169288 or rs1260326 were again replicated and the overall associations in the combined study were beyond the GWAS significant level (GLM *P*=2.2 × 10^−23^, 1.5 × 10^−12^ and 3.1 × 10^−16^, respectively, Table 2 and Supplementary Fig. 6A–C). The association of rs11068574 was again not replicated (GLM *P*=0.093, Table 2). All of the SNPs showed success rates of more than 0.96 and did not show departure from Hardy–Weinberg Equilibrium in each of the three studies (*χ*^2^-test *P*≥0.0019) and the combined study (*χ*^2^-test *P*≥0.045).

### Explained effects by the combination of the significant SNPs

**Figure 2:**
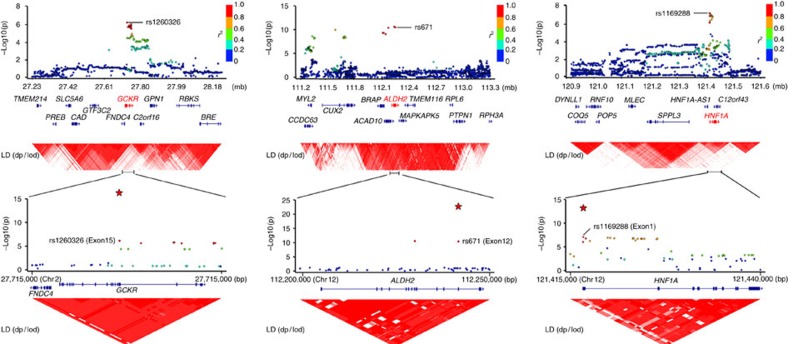
Regional plots of the three regions significantly associated with the serum AAT levels. The chromosomal positions and *P* values for SNPs in the three significant regions are shown accordingly. Brightness of the red colour in LD blocks corresponds to the strength of LD. The red stars in the lower panels represent the associations in the combined study (*n*=9,359).

All of the three markers showing significant associations are located in exonic regions of the genes and induce amino-acid alterations of the proteins (Fig. 2 and Table 2). We did not find heterogeneity between GWAS results and replication results for these three markers (Cochran's Q test *P*≥0.14). When we evaluated interactions between any pairs of these three SNPs, we did not find strong interactions on serum AAT levels (GLM *P*≥0.038). When we analysed the association between the number of susceptibility alleles of these three SNPs and serum AAT levels using GLM, the association showed a *P* value of 1.5 × 10^−48^ (Supplementary Fig. 6D). These three SNPs were evaluated to explain 1.95% of total variance of the serum AAT levels (Supplementary Table 2).

### Functional annotation search for causative SNPs

We picked up all of the variants in strong LD (*r*^2^>0.8) with the three SNPs and performed functional annotation search of these SNPs by HaploReg^17^ (Supplemetary Table 3). None of the other missense mutations in the three genes were in LD with the three SNPs. The block of the five SNPs in strong LD with rs671 included *ACAD10* and *BRAP* as well as *ALDH2* (Supplementary Fig. 5 and Supplementary Table 3). *ALDH2* was the only gene among the three genes in the region whose functional relevance to AAT was reported^18^,^19^ and which contained SNPs with functional annotations in hepatic cells (Supplementary Table 3). *HNF1A* was shown to be an important expression regulator of AAT^20^, and the two SNPs in strong LD with rs1169288 also resided in the *HNF1A* locus. The block of the 14 SNPs in strong LD with rs1260326 contained only the *GCKR* locus and was more than 46.5 kbp away from the adjacent gene, *C2orf16* (Supplementary Table 3 and Fig. 2). Thus, it is reasonable to regard *ALDH2*, *HNF1A* and *GCKR* as the responsible genes for the ssociations with levels of AAT. Notably, all of the three genes are expressed in the liver and are reported to be associated with metabolic phenotypes including impairment of insulin secretion^21^, lipid profiles^22^,^23^, risk of type 2 diabetes mellitus^24^ and cardiovascular diseases^25^ and metabolic syndrome pathway^26^.

### Analysis of HNF1A and GCKR conservations

While the functional importance of amino-acid alteration of ALDH2 by rs671 is well established (described later), functional impacts of amino-acid alteration by rs1169288 and rs1260326 on HNF1A and GCKR are not fully known except for some reports^27^. Polyphen-2 software^28^ did not suggest deleterious change brought about by the two SNPs. Thus, we analysed conservation of these amino-acid residues across vertebrates. As a result, isoleucine altered by rs1169288 was highly conserved in vertebrates (Supplementary Table 4), supporting the impact of rs1169288. Proline induced by rs1260326 rather than leucine was conserved among vertebrates (Supplementary Table 4), suggesting the importance of the C allele of rs1260326.

### Exclusion of possibility of confounded by CRP

It might be noted that *HNF1A* and *GCKR* are known susceptibility loci to CRP levels^26^, which are correlated with the levels of AAT. We confirmed no association between the residuals of AAT and serum CRP levels, indicating that the associations in these two regions were not dependent on the associations between CRP levels and the two regions (GLM *P*=1.0, Supplementary Fig. 7).

### Expression correlations between *SERPINA1* and the three genes

We analysed whether these three gene expressions are associated with expressions of *SERPINA1* using gene expression database in the Japanese population^29^ to infer the relationship between AAT and the three proteins encoded by these three genes. As a result, the expressions of *ALDH2, GCKR* and *HNF1A* were significantly associated with that of *SERPINA1* or the transcript variant of *SERPINA1* (Student's *t* distribution test *P*=5.6 × 10^−15^, Spearman's rank sum coefficient (*r*_s_) =0.43, *P*=3.7 × 10^−6^, *r*_s_=0.26 and *P*=1.7 × 10^−8^, *r*_s_=0.31, respectively, Supplementary Fig. 8). These results may suggest a molecular network among these genes and support the association between AAT and *GCKR* as well as those between AAT and *ALDH2* or *HNF1A*.

### Detailed analysis of *ALDH2*

Next, we concentrated on the associations observed in *ALDH2*, the most significant locus with previously reported functional analyses. ALDH2 dehydrogenates acetaldehyde that is produced by various processes and inhibits the anti-elastase activity of AAT^19^. As ethanol is dehydrogenated into acetaldehyde, ALDH2 is also involved with alcohol consumption. The A allele of rs671 is known to decrease ALDH2 activity and lower alcohol tolerance^18^,^23^,^30^. In fact, rs671 is very strongly associated with alcohol consumption in the current study (Student's *t* distribution test *P*=2.2 × 10^−248^). As alcohol consumption is known to affect AAT levels presumably via acetaldehyde^31^, the association of *ALDH2* might be explained by altering alcohol consumption due to rs671. In fact, conditioning on alcohol consumption greatly diminished the association of rs671 (after conditioned; GLM *P*=9.2 × 10^−8^). We still found a substantial association after conditioning, suggesting further mechanisms aside from altering alcohol drinking by rs671. In contrast, conditioning on alcohol did not alter the associations of rs1260326 and rs1169288 (GLM *P*=3.0 × 10^−16^ and 6.6 × 10^−12^, respectively). We also confirmed that no novel susceptibility markers appeared in GWAS using alcohol consumption as an additional covariate.

### Interaction between rs671 in *ALDH2* and alcohol consumption

**Figure 3:**
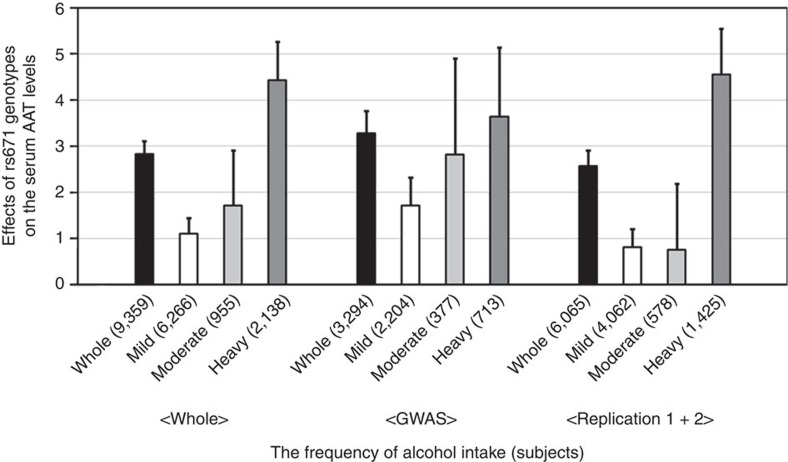
The interaction between the rs671 genotypes and alcohol drinking on the serum AAT levels. The effects of rs671 on serum AAT levels in linear regression analysis are shown in the groups subdivided into three groups based on alcohol intake. Error bars represent standard errors.

As rs671 has interactive effects with alcohol consumption on phenotypes including metabolic trait^32^,^33^, we hypothesized that rs671 and alcohol would show an interactive effect on serum AAT levels as well. As a result, we identified a significant interaction between rs671 genotypes and alcohol intake (GLM *P*=2.2 × 10^−4^, Fig. 3). rs671 A allele showed a trend of increase in its effect on AAT in those who were heavily drunk (Fig. 3). The results of other interactions are shown in Supplementary Table 5. While we also found an interaction between rs671 and smoking (GLM *P*=6.8 × 10^−4^, Supplementary Table 5 and Supplementary Fig. 9), this seems to be explained by strong correlation between alcohol consumption and smoking (*r*_s_=0.41, Student's *t* distribution test *P*=7.2 × 10^−380^). In fact, when the interaction between rs671 and smoking was evaluated by conditioning on the interaction between rs671 and alcohol intake, the effect was not significant (GLM *P*=0.16). When we assessed the interactions between rs1260326 in *GCKR* or rs1169288 in *HNF1A* and alcohol or smoking, no significant interactions were observed (Supplementary Table 6).

### Association between FEV1.0 and the three SNPs

Next, we evaluated whether these genetic components associated with AAT levels in the current study are associated with lung function alone or in combination because patients with AATD presented with COPD in their youth. At first, we did not find a significant association between serum AAT levels and FEV1.0 (Supplementary Fig. 10a). While rs1260326 and rs1169288 did not show significant associations with FEV1.0, rs671 showed a negative association with FEV1.0 (GLM *P*=4.5 × 10^−5^, Supplementary Fig. 10b). We observed this association even after conditioning for AAT levels (GLM *P*=6.1 × 10^−5^, Supplementary Fig. 11a), suggesting the association of ALDH2 unrelated to AAT. Therefore, the sum of susceptibility alleles of these three SNPs showing a suggestive negative association with FEV1.0 can be explained by the negative association of rs671 (GLM *P*=0.0063 and 0.0086, before and after conditioning on AAT, Supplementary Figs 10C and 11B). As smoking has a big impact on the development of COPD in patients with AATD^34^, we evaluated the association between FEV1.0 and rs671 based on smoking status. The negative association was found to be observed mainly in the non-smoking population (GLM *P*=8.1 × 10^−5^, Supplementary Fig. 12).

### Comparison of associated SNPs between Japanese and Europeans

Finally, to evaluate the SNPs significantly associated with the serum AAT level in the European population, we searched the effects in the current study of the 14 SNPs whose significant or suggestive associations were reported in the European GWAS. Nine out of the 14 SNPs, including rs17580 (PI S) and rs28929474 (PI Z), showed no or very small minor allele frequencies (minor allele frequency (MAF) ≤0.006). The results of the remaining five SNPs are shown in Table 3. Although rs2896268 showed an association with the comparable effect size to the European study(GLM *P*=2.4 × 10^−4^, Table 3), the other four markers did not show a trend of association in spite of high minor allele frequencies (GLM *P*≥0.42, MAF≥0.31, Table 3).

## Discussion

The current GWAS and the following two-staged replication study identified a total of three missense SNPs significantly associated with serum AAT levels in the *ALDH2* and *HNF1A* loci on chromosome 12 and the *GCKR* locus on chromosome 2. *ALDH2* and *HNF1A* have been reported to be functionally associated with AAT. Significant correlations of gene expression between *SERPINA1* and all of the three genes support functional relevance of these genes to AAT.

While the SNPs in LD with rs671 contained two other genes and *BRAP* was associated with metabolic syndrome^35^ as well as *ALDH2*, the deleterious effect of rs671, the strongest association of the *ALDH2* region and the functional involvement of ALDH2 with AAT activity suggest *ALDH2* as a responsible gene. rs671 is known to strongly affect alcohol consumption by making people get drunk easily and be light drinkers through decreasing ALDH2 activity^23^,^30^. The decrease of ALDH2 activity is attributed to the impaired binding affinity to coenzyme, NAD^+^ (ref. 36) and triggers the facial flushing caused by alcohol intake^37^,^38^ and local inflammatory diseases^39^,^40^,^41^. The heterozygous rs671 shows 6% of the normal activity, and homozygous variant shows almost negative activity^18^. Although the alteration of alcohol consumption by rs671 seemed to explain more than half of the association in the *ALDH2* region, the substantial association remained after conditioning on alcohol consumption. The significant interaction between rs671 and alcohol intake revealed the stronger association of rs671 in heavy drinkers. Functional annotation analysis revealed DNaseI hypersensitivity site (DHS) and enhancer activity in liver-derived cells at rs4646776 in intron 8 of *ALDH2*, in almost complete LD with rs671 (Supplementary Table 3). Thus, (1) alteration of alcohol consumption by rs671, (2) interaction between rs671 and alcohol consumption and (3) potential transcriptional alteration of *ALDH2* induced by a haplotype of rs671 and rs4646776 may explain the association in this region.

While functional importance of rs1169288 on HNF1A protein is not known and *in silico* analysis using Polyphen-2 suggests a benign change brought about by this SNP, conserved search suggested the importance of this SNP. In fact, rs1169288 has been reported for its association with impairment of insulin secretion^21^ and risk of type 2 diabetes mellitus^24^ and cardiovascular diseases^25^ in community-dwelling populations. The functional annotation search revealed active promoter effects in liver-derived cells at rs1169288 and the other two SNPs in LD with rs1169288 (Supplementary Table 3). It also showed DHS and binding of transcription factor in hepatic cells at rs1169288 and one of the two SNPs. A conformational or transcriptional alteration of HNF1A via rs1169288 and/or SNPs in LD with rs1169288 would lead to modulation of the regulatory effects of HNF1A on *SERPINA1* expression and explain the association in the *HNF1A* region.

While *in silico* analysis using Polyphen-2 did not result in damaging effect brought about by rs1260326, a recent *in vitro* analysis reported that the C allele of rs1260326 leads to increased glucokinase activity in the liver and resultant increase of triglyceride and decrease of glucose levels^27^. Functional search revealed enhancer activity and DHS in liver-derived cells at rs1260326 (Supplementary Table 3). It also showed that three other SNPs in LD with rs1260326 have strong enhancer activity and transcription factor binding in hepatic cells. rs1260326 was reported to be associated with metabolic traits including HOMA-IR^42^, lipid profiles^22^, NAFLD and NASH with fibrosis^43^. Conservation of proline across vertebrates may suggest advantage of increased glucokinase activity and AAT levels. An increased glucokinase activity and potential transcriptional alteration in the liver due to rs1260326 and other SNPs in LD with rs1260326 would explain the association between AAT and the *GCKR* region.

It should be noted that all of the three genes are associated with metabolic phenotypes and are expressed in the liver. As AAT is predominantly produced in the liver, the associations between these three genes and AAT suggest that these genes modulate AAT levels mainly in the liver. When we analysed whether the three SNPs display *cis* associations with expressions of *ALDH2*, *GCKR* and *HNF1A*, respectively, or *trans* associations with expression of *SERPINA1*, we did not observe significant associations. Since functional annotation analysis suggested enhancer activity and/or DHS at the three SNPs and/or variants in strong LD with the SNPs in liver-derived cells, we might detect significant *cis* or *trans* effects of the SNPs in expression data from liver-derived cells. As the three variants are missense variants of genes predominantly expressed in the liver in which AAT is mainly produced, it is also likely that these three SNPs influence serum AAT levels through conformational change derived from amino-acid alterations, especially rs671.

While AAT is an enzyme controlling inflammation by inhibiting proteases like the neutrophil elastase, recent studies have revealed a broad range of AAT functions including inflammation, apoptosis, cellular senescence, lipid metabolism and proteostasis. Thus, the associations between AAT and the three loci related to metabolic phenotypes suggest the involvement of AAT with the metabolic syndrome-related pathway beyond its function as an acute inflammatory marker.

While patients with AATD present with COPD, we did not find a significant association between AAT levels and FEV1.0. In addition, we did not find significant associations between FEV1.0 and genotypes except for rs671. As the A allele of rs671 is associated with increased AAT levels and decreased FEV1.0, the association between rs671 and FEV1.0 does not match with the relationship between AATD and COPD. We did not observe a significant positive association between rs671 and FEV1.0 even in the smoking population (Supplementary Fig. 12). Further replication study is necessary to confirm the negative association of rs671 A allele with FEV1.0 independent of AAT. The poor translation of genetic associations with AAT into lung function was also observed in the European study^13^. As lung functions are finely regulated by multiple complexed mechanisms including smoking and systemic or local inflammation, alterations of lung function derived from these three genetic components associated with serum AAT levels might be limited and difficult to identify. Another possibility is that serum AAT levels do not precisely represent AAT levels in the lung. Uncovering mechanisms regulating AAT levels in the lung and appropriate modelling for lung AAT levels would lead to elucidation of the importance of genetic components associated with AAT levels on lung function.

The three SNPs were not reported in the previous European GWAS^13^. As the A allele of rs671 was frequently observed in Asian populations and is rarely seen in European populations, the lack of the association between rs671 and AAT in the European study seems appropriate. In addition, all of the SNPs in strong LD with rs671 in the Japanese population showed very small allele frequencies in European population (Supplementary Table 3). In contrast, rs1169288 and rs1260326 have comparable allele frequencies in Asians and Europeans (0.539 versus 0.283 and 0.490 versus 0.600, respectively, Hapmap phase II data). There are several possibilities to explain the discrepancy of the two associations between the two populations. First, the lack of power (*n*=1,392) or overrepresentation of asthmatic patients in the previous European GWAS may explain the lack of significant associations in the Europeans. Second, there might be other causative variants that are in LD with the two SNPs that can specifically be found in the Japanese and not in the European population due to the different LD structures between populations. However, no SNPs in strong LD with the two SNPs in Japanese showed highly different allele frequencies from the two SNPs in European population (Supplementary Table 3). As the two SNPs were associated with multiple phenotypes in European populations and the functional importance of rs1169288 was experimentally shown, the first possibility seems more likely. It is reasonable to perform quantitative trait linear regression analysis of AAT levels in a large-scale healthy European population.

The four SNPs in the *SERPINA* gene clusters showing significant associations in the European study, namely, rs2736887, rs926144, rs4905179 and rs11621961, did not show evidence of associations in the current study. As the associations in the European population were shown to largely depend on PI Z and PI S and these two variants were not found in the current participants, the lack of associations of these four SNPs in the current study seems reasonable. We found a suggestive association of rs2896268 with a comparable effect size to the European population. rs2896268 showed an association independent of PIS and PIZ in the Europeans, suggesting a common mechanism in the regulation of *SERPINA1* expression beyond ethnicity that is independent of PI S and PI Z.

As our results showed an association of the Asian-specific variant and associations of the common variants in *GCKR* and *HNF1A* with serum AAT levels, further replication studies and functional experiments recruiting other Asian populations and Europeans are necessary.

The increase of serum CRP levels is an independent risk factor of developing metabolic syndrome^44^, diabetes mellitus^45^, stroke and cardiac infarction^46^, and therefore it is reasonable to quantify AAT levels in a prospective manner to test AAT as the possible independent risk factor for metabolic syndrome, diabetes mellitus or stroke. In addition, Mendelian randomization approach^47^,^48^ to test the association between these three markers and developing metabolic syndrome, diabetes mellitus or stroke would clarify the direct or indirect function of AAT beyond acute phase inflammation marker.

## Methods

### Study subjects

This study was performed as a part of The Nagahama Prospective Genome Cohort for Comprehensive Human Bioscience (the Nagahama Study)^14^. The participants of The Nagahama Study were recruited from the general population living in Nagahama City, a largely rural city of 125,000 inhabitants in Shiga Prefecture, located in the centre of Japan. Persons aged 30 to 74 years, living independently in the community and with no physical impairment or dysfunction, were enrolled and detailed questionnaires were answered by the participants. Among 9,804 participants recruited from 2008 to 2010, 9,761 subjects were registered for our analyses excluding pregnant women (*n*=43). All study procedures were approved by the ethics committee of Kyoto University Graduate School of Medicine and the Nagahama Municipal Review Board. Written informed consent was obtained from all participants.

### Genotyping

Among the total of 9,761 samples, genome scanning was conducted on 3,693 samples by using one of or combinations of human hap610K quad array (hap610k), human omni 2.5M-4 array (2.5M-4), human omni 2.5M-8 array, (2.5M-8) human omni 2.5s array (2.5M-s), human exome (Exome) and human core exome (CoreExome) (Illumina, San Diego, CA, USA). The detailed distribution of samples for genome-scanning arrays are shown in Supplementary Table 7. The remaining 4,023 and 2,042 DNA samples were used for the replication studies 1 and 2, respectively. The details of sample selection are written in Supplementary Note 1 and Supplementary Fig. 2. The genotyping of the SNPs in the replication studies was conducted by TaqMan assay (Applied Biosystems).

### Quality control for the genotyping data

In the genome scanning of 3,693 subjects, 57 individuals were excluded with a low call rate less than 0.90, 0.95 and 0.99 in the hap610k, 2.5M-4 and other plat forms, respectively. Seven individuals were excluded due to outliers from Asian clusters by principal component analysis with HapMap Phase 2 release 28 JPT data set as reference (EIGENSTRAT ver. 2.0, Supplementary Fig. 3)^49^ and a total of 316 subjects were excluded due to high degrees of kinship (Pi-hat greater than 0.35, PLINK ver. 1.07)^50^. Nineteen samples were excluded because they lack information of smoking status.

SNPs with a call rate less than 0.99, MAF below 0.01 or showing departure from HWE (*χ*^2^-test *P*<1.0 × 10^−6^) were excluded from the analyses. As a result, 267,882 SNPs remained for the following genotype imputation. The details of these quality check procedures are shown in Supplementary Fig. 2.

### Genotype imputation

Genotype imputation was conducted using a standard procedure with MACH ver. 1.0.16 software^51^ using East Asian panel in the 1000 Genome project as a reference. Imputed SNPs with MAF below 0.01 or R-square (RSQ) below 0.3 were removed from the subsequent association analysis. In total, 6,569,727 SNPs were used in the following GWAS on the serum AAT level.

### Quantification of AAT and CRP levels

The serum levels of AAT and high-sensitive CRP (hs-CRP) were measured by nephelometry on BN II (R) Nephelometer (Siemens Healthcare Diagnostics, Munchi, Germany) using N-antiserum to human alpha1-antitrypsin kit and N-latex CRP II kit (the same company), respectively. These assays were performed at SRL Laboratories (Tokyo, Japan) and were commercially available. The intra- and inter- assay coefficients of variation of the AAT levels were 1.07–1.67% and 1.02–1.31%, respectively.

### Evaluation of LD

LD between markers were evaluated by PLINK^50^ software or Haploview software^52^.

### Heterogeneity of the genetic studies

Heterogeneity among GWAS and the two replication studies was assessed by Cochran's Q test for the three SNPs showing significant associations beyond the GWAS significant level in the combined study.

### Variance explained by the significantly associated SNPs

Variance that was explained by the three SNPs significantly associated with AAT levels was calculated based on the following formula:


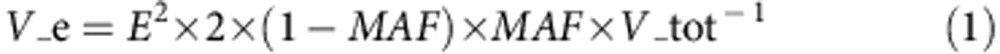


Where *V_*e is an explained variance by an SNP, *E* is an effect size of the SNP, *MAF* is minor allele frequency of the SNP and *V_*tot is total variance of AAT.

### Functional annotation and conservation of amino acids

We used HaploReg^17^ and analysed whether SNPs in strong LD with the top SNPs have functional effects including promoter or enhancer activity, DHS and binding of transcription factor. *In silico* analysis of assessing effects of amino-acid alteration induced by the associated markers were performed by Polyphen-2 software^28^. Conservation of amino-acid sequences including amino acids altered by the associated markers was analysed by using UCSC Genome Browser on Human Feb. 2009 (GRCh37/hg19) Assembly.

### Gene expression analysis

The expression data were obtained by the database of whole peripheral blood in the Japanese population^29^ (Human Genetic Variation Browser: http://www.genome.med.kyoto-u.ac.jp/SnpDB/). We used the expression data of *SERPINA1* (Target ID: NM_001002236), a transcriptional variant of *SERPINA1* (*SERPINA1_Var*) (Target ID: ENST_00000402629), *GCKR* (Target ID: NM_001486), *ALDH2* (Target ID: NM_000690) and *HNF1A* (Target ID: NM_000545). We assessed the correlation between gene expressions by Spearman's rank correlation coefficient. The *P* values for the significance of the coefficients were calculated by Student's *t* distribution test. The associations between gene expressions and genotypes were analysed by generalized linear regression model adjusted by sex, age, BMI, smoking status, the experimental day and the logarithm of hs-CRP. The smoking status consists of three categories, namely, never-smoker, ever-smoker and current-smoker.

### The interactions between correlates on the serum AAT level

The interactions between SNPs were analysed in linear regression model with the use of the same covariates described in the Statistical analysis section. The interactions between each of the three SNPs and alcohol consumption consisting of three categories (Heavy: >3days week^−1^, Moderate: 2–3days week^−1^, Mild: <2days week^−1^) or Brinkmann index, defined by smoking pack day^−1^ multiplied by year which reflects the lifetime smoking exposure^53^, were also analysed. We also analysed interaction between rs671 and age, sex, BMI or CRP.

### Statistical analysis

GWAS and replication studies on the serum AAT levels were conducted by GLM adjusting for the recruiting year, age, sex, BMI, Brinkman index and the common logarithm of hs-CRP. These covariates used in the analyses were selected from non-genetic factors considered in another GWAS report (age, sex and the smoking status)^13^ and based on the perceptions on AAT as an acute-phase inflammation marker^2^. Analysis adding alcohol consumption was also performed. The cigarette smoking and alcohol consumption history was obtained from the questionnaire. When we conducted conditional GWAS to find other candidate SNPs associated with serum AAT level independent of each of the associated SNPs, we added the SNP genotype into the former liner model as a covariate. Correlation between smoking or rs671 and alcohol consumption was assessed by Spearman's rank sum correlation coefficient.

All statistical calculations were conducted using the free software R (http://www.r-project.org/)^54^ or PLINK. *P* values less than 5.0 × 10^−8^ were considered to satisfy the genome-wide significance in the combined study. Otherwise, *P* value less than 0.05 after Bonferroni's correction was regarded as significant.

## Additional information

**How to cite this article:** Setoh, K. *et al*. Three missense variants of metabolic syndrome-related genes are associated with alpha-1 antitrypsin levels. *Nat. Commun.* 6:7754 doi: 10.1038/ncomms8754 (2015).

## Supplementary Material

Supplementary Information Supplementary Figures 1-12, Supplementary Tables 1-7 and Supplementary Note 1

## Figures and Tables

**Table 1:** The characteristics of study subjects.

	GWAS	Replication 1	Replication 2
Number	3,294	4,023	2,042
Sex (female ratio) (%)	66.5	67.0	68.8
Age* (year)	52.3±14.1	55.3±12.6	53.1±12.9
AAT* (mg dl^−1^)	133.0±19.4	128.5±17.6	135.4±19.0
BMI* (kg m^−2^)	22.3±3.2	22.3±3.3	22.3±3.3
Brinkman index*	164.6±319.8	167.2±325.5	149.4±307.6
Alcohol intake† (heavy/moderate/mild)	713/377/2,204	959/379/2,685	466/199/1,377
High-sensitive CRP* (ng ml^−1^)	922.1±3261	937.6±4108	786.5±2548

AAT, Alpha-1 antitrypsin; BMI, body mass index; CRP, C-reactive protein; GWAS, genome-wide association studies.

^*^The mean±s.d.

^†^Heavy: >3 days week^−1^, moderate: 2–3 days week^−1^, mild <2 days week^−1^.

**Table 2:** Association studies on AAT serum level in the Nagahama Study.

**Table 3:** The associations between SNPs reported in the European GWAS and serum AAT levels in the Japanese population.

SNP	Chr	Position	Gene	Ref/var	Location	The European study				The current study			
						N	VAF	Effect	*P*	N	VAF	Effect	*P*
rs2736887	14	94812980	*SERPINA2A/SERPINA6*	G/C	Intergenic	1,392	0.185	7.1	2.5 × 10^−13^	3,294	0.576	0.34	0.44
rs926144	14	94813402	*SERPINA2A/SERPINA6*	A/G	Intergenic	1,392	0.186	7.1	2.7 × 10^−13^	3,294	0.549	0.29	0.51
rs4905179	14	94795492	*SERPINA6*	A/G	Upstream	1,392	0.180	6.8	1.2 × 10^−12^	3,294	0.476	0.35	0.42
rs11621961	14	94769476	*SERPINA6*	C/T	Downstream	1,392	0.355	5.2	1.4 × 10^−11^	3,294	0.310	0.37	0.44
rs2896268	14	94865708	*SERPINA1*	A/C	Upstream	5,569	0.495	1.3	4.1 × 10^−5^	3,294	0.502	1.61	2.4 × 10^−4^

Chr, chromosome; *N*, number of subjects; *P, P* value calculated by generalized linear regression model; ref/var: reference allele/variant allele; VAF, variant allele frequency.

Effects in European sutdy are converted to mg dl^−1^.
