# Supplementary material for: Three missense variants of metabolic syndrome-related genes are associated with alpha-1 antitrypsin levels
Source: Nat Commun. 2015 Jul 15;6:7754. doi: 10.1038/ncomms8754 (PMC4518310; doi:10.1038/ncomms8754)
Supplement: Supplementary Information — Supplementary Figures 1-12, Supplementary Tables 1-7 and Supplementary Note 1 [file ncomms8754-s1.pdf]

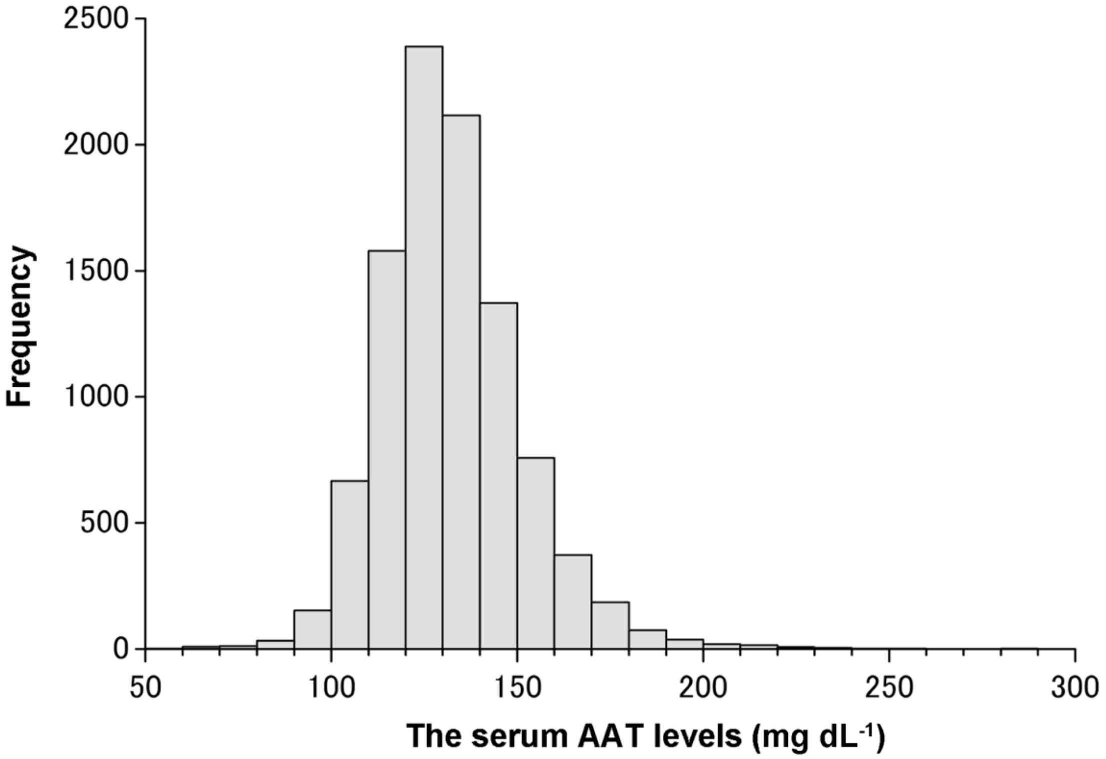

Supplementary Figure 1 | Distribution of serum AAT levels

A histogram of serum AAT levels is indicated.

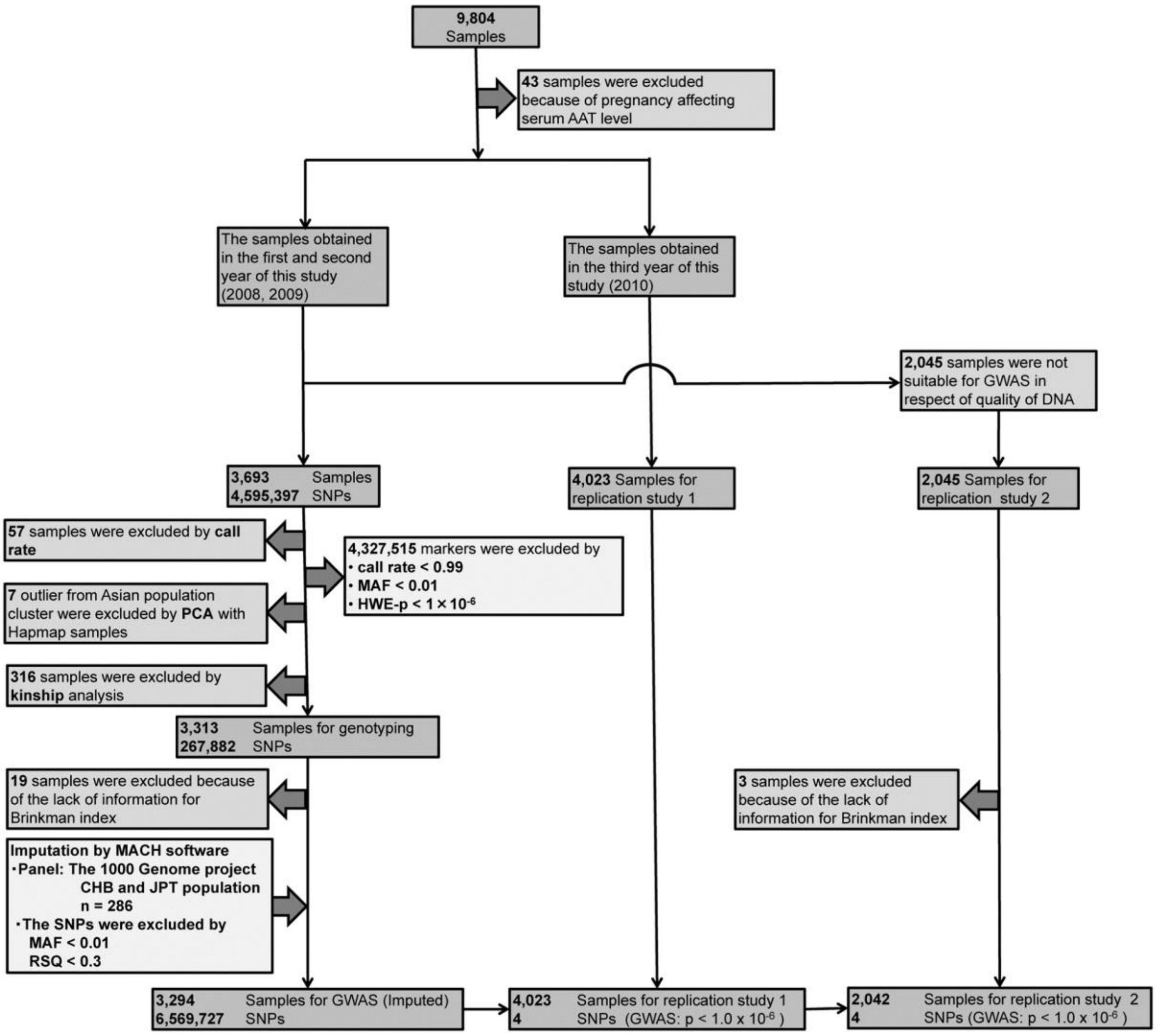

Supplementary Figure 2 | A flowchart of sample selection and quality control in the current study

A schematic view of sample selection and quality control of samples and SNPs in the current study is indicated.

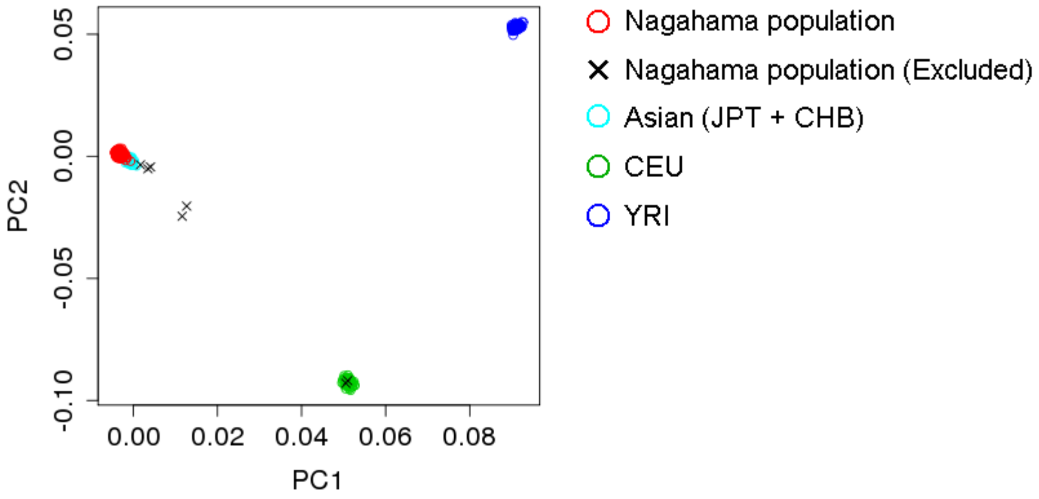

Supplementary Figure 3 | A result of principal component analysis of GWAS data

A result of principal component analysis is indicated according to principal components 1 and 2. The 7 samples shown as black crosses were excluded as outliers.

PC:principal component, JPT:Japanese in Tokyo, CHB:Chinese in Beijing, CEU:Utah residents with Northern and Western European ancestry, YRI:Yoruba in Ibadan

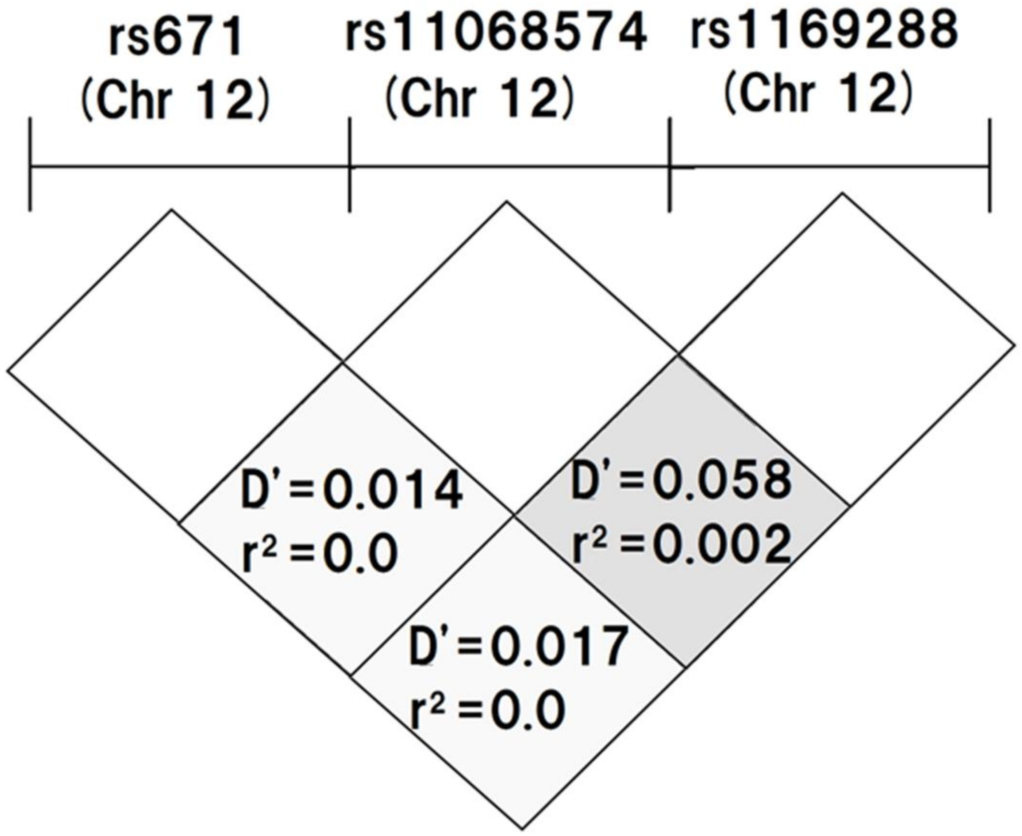

Supplementary Figure 4 | Linkage disequilibrium among the three SNPs on chromosome 12

The LD structure of the three SNPs on chromosome 12 showing associations with AAT in GWAS is indicated.

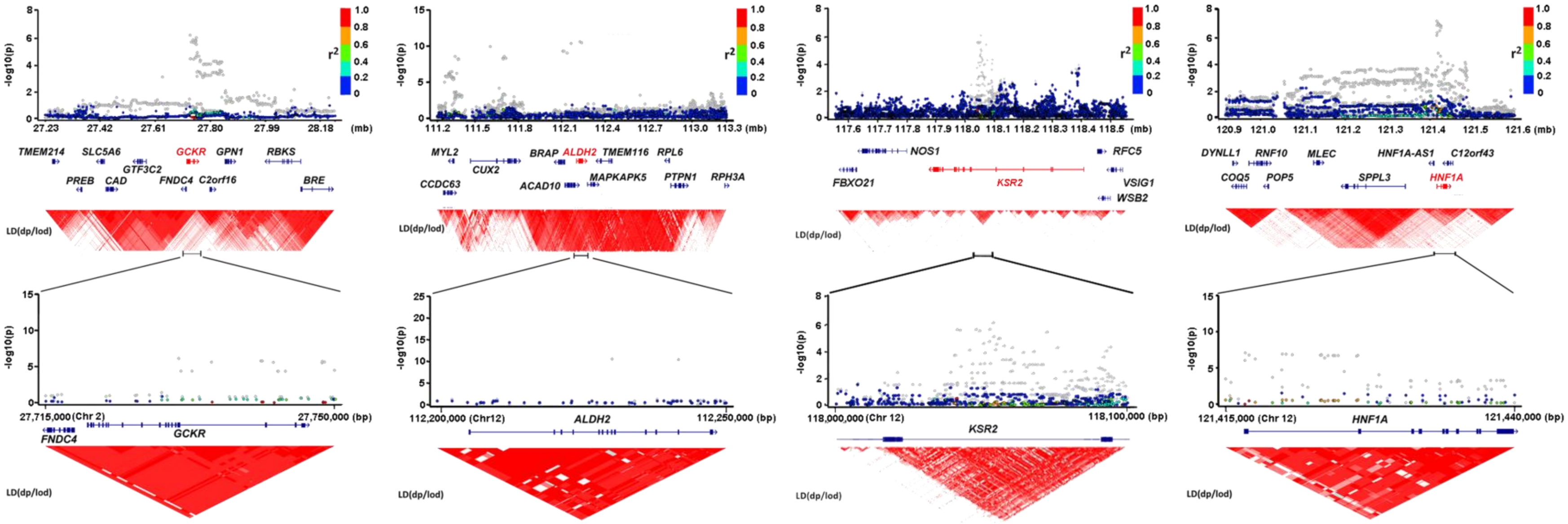

Supplementary Figure 5 | Conditional analyses reveal single association in each of the four regions selected for the replication studies

Each association is plotted according to its position (X axis) and its strength of association (Y axis). Gray and colored circles in each region indicate associations before and after conditioning on the SNP selected for replication studies, respectively.

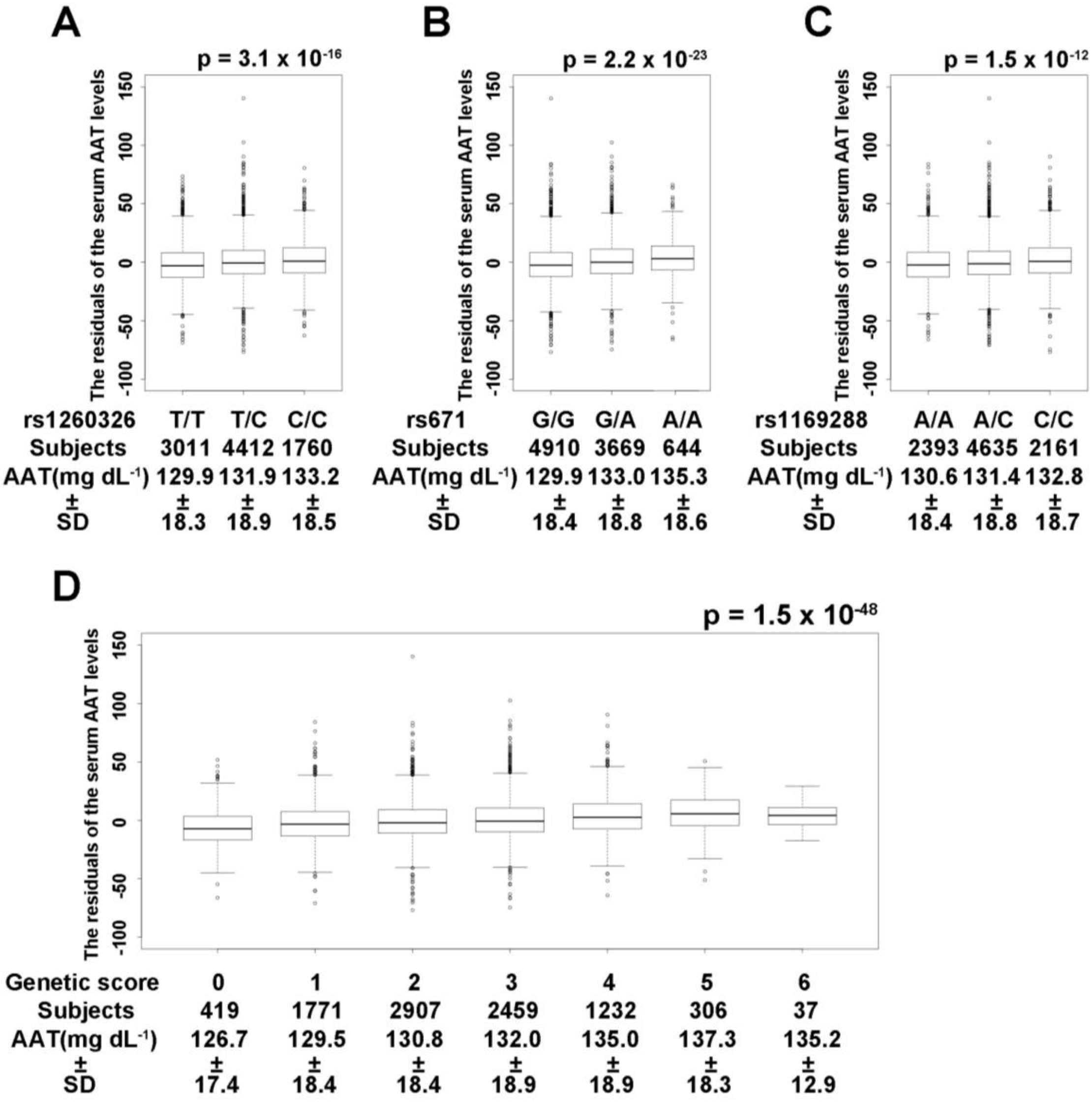

Supplementary Figure 6 | The associations between serum AAT levels and the three SNPs alone or in combination

The box plots showing the associations between AAT and A)rs1260326, B)rs671, C)rs1169288 or D) the number of the risk alleles of the three SNPs are indicated. The upper and lower boundaries of the boxes represent the third and first quartiles, respectively. The horizontal lines represent the medians. The whiskers represent more or less than 1.5 times of the interquartile range.

P: p-value calculated by generalized linear regression model.

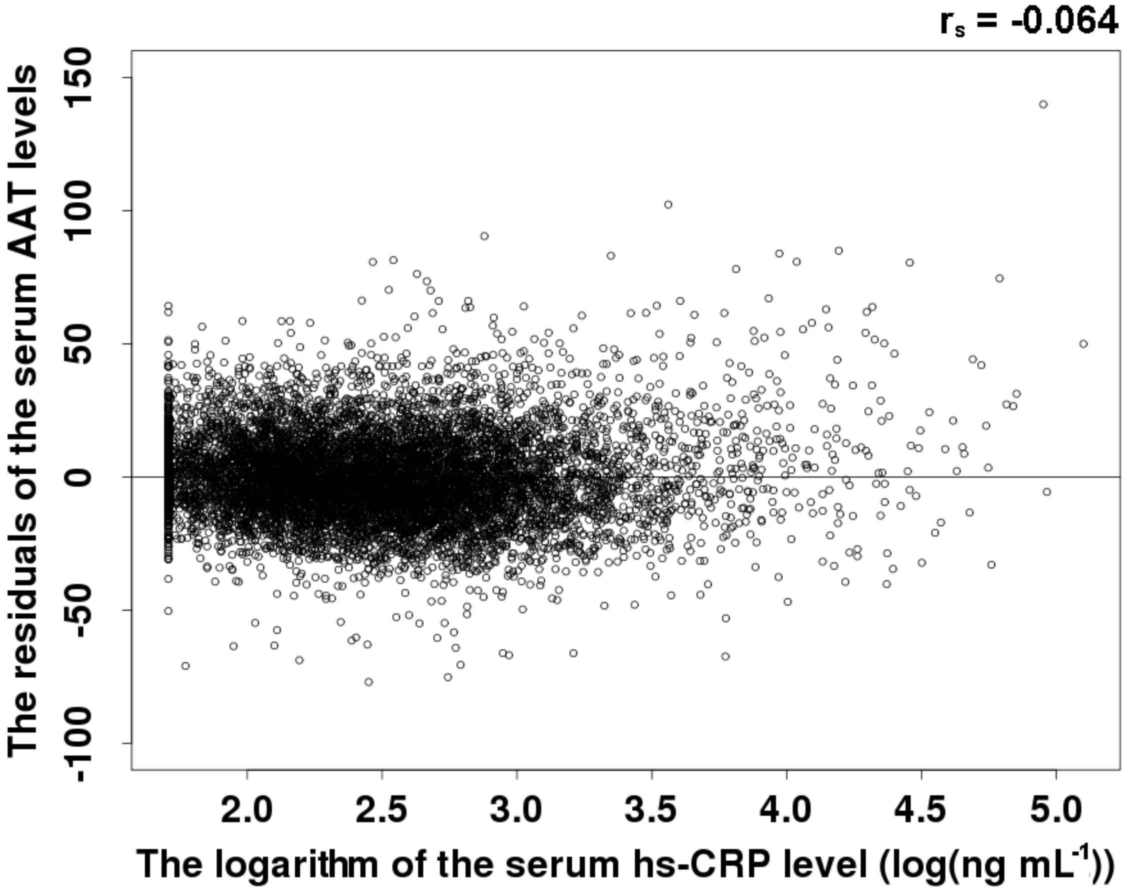

Supplementary Figure 7 | Lack of confounding associations between CRP levels and residuals of generalized linear regression analysis

The association between hs-CRP and the residuals of AAT in the generalized linear regression model is indicated.

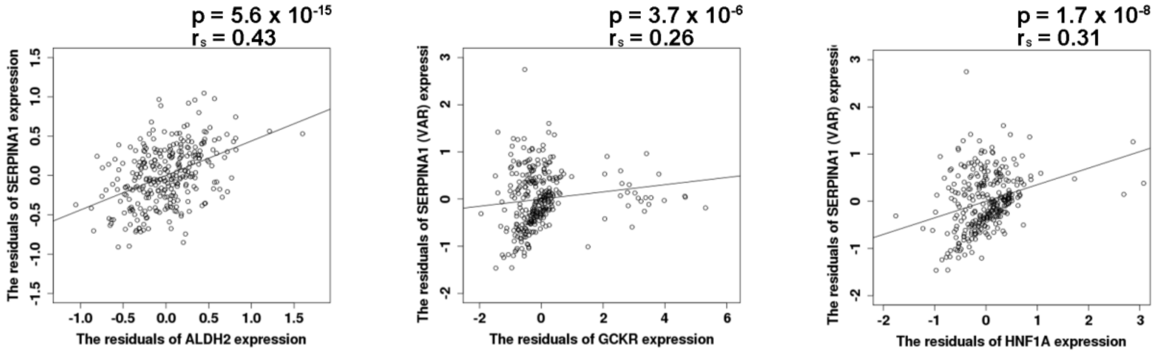

Supplementary Figure 8 | The associations of gene expressions between *SERPINA1* and *ALDH2, GCKR* or *HNF1A*

The data is based on the data of the Human Genetic Variation Browser.

P: p-value calculated by Student's t-distribution test. r_s: Spearman's rank sum coefficient.

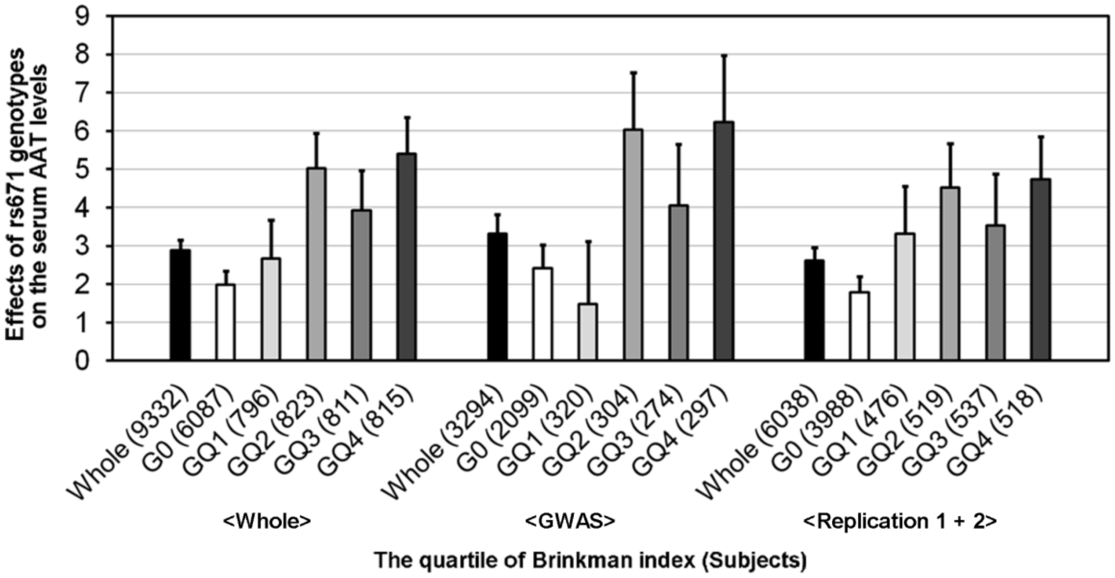

Supplementary Figure 9 | Interaction between smoking and rs671

Interactive effect sizes between smoking and rs671 are shown in the combined study, GWAS and replications 1 and 2. Error bars indicate standard errors. G0: non-smokers, GQ1-4: smokers quartile according to Brinkman index.

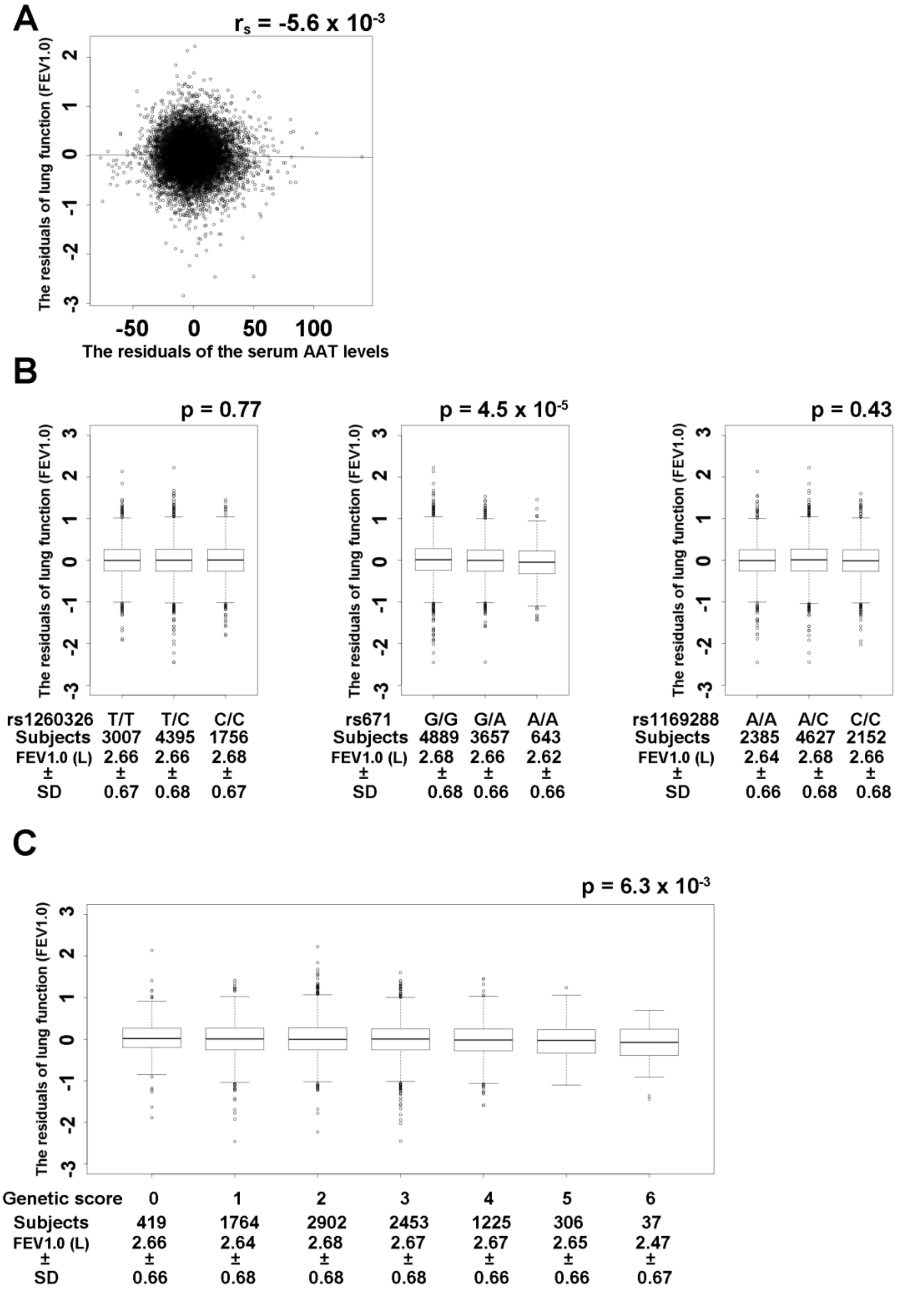

Supplementary Figure 10 | Associations between lung function and serum AAT levels or the three variants associated with serum AAT levels

A) The lack of association between AAT and FEV1.0 in the current study is indicated.

The associations between AAT and the three SNPs (B) or the number of risk alleles of the three SNPs(C) are indicated.

r_s: Spearman's rank sum coefficient. P: p-value calculated by generalized linear regression model.

The upper and lower boundaries of the boxes represent the third and first quartiles, respectively. The horizontal lines represent the medians. The whiskers represent more or less than 1.5 times of the interquartile range.

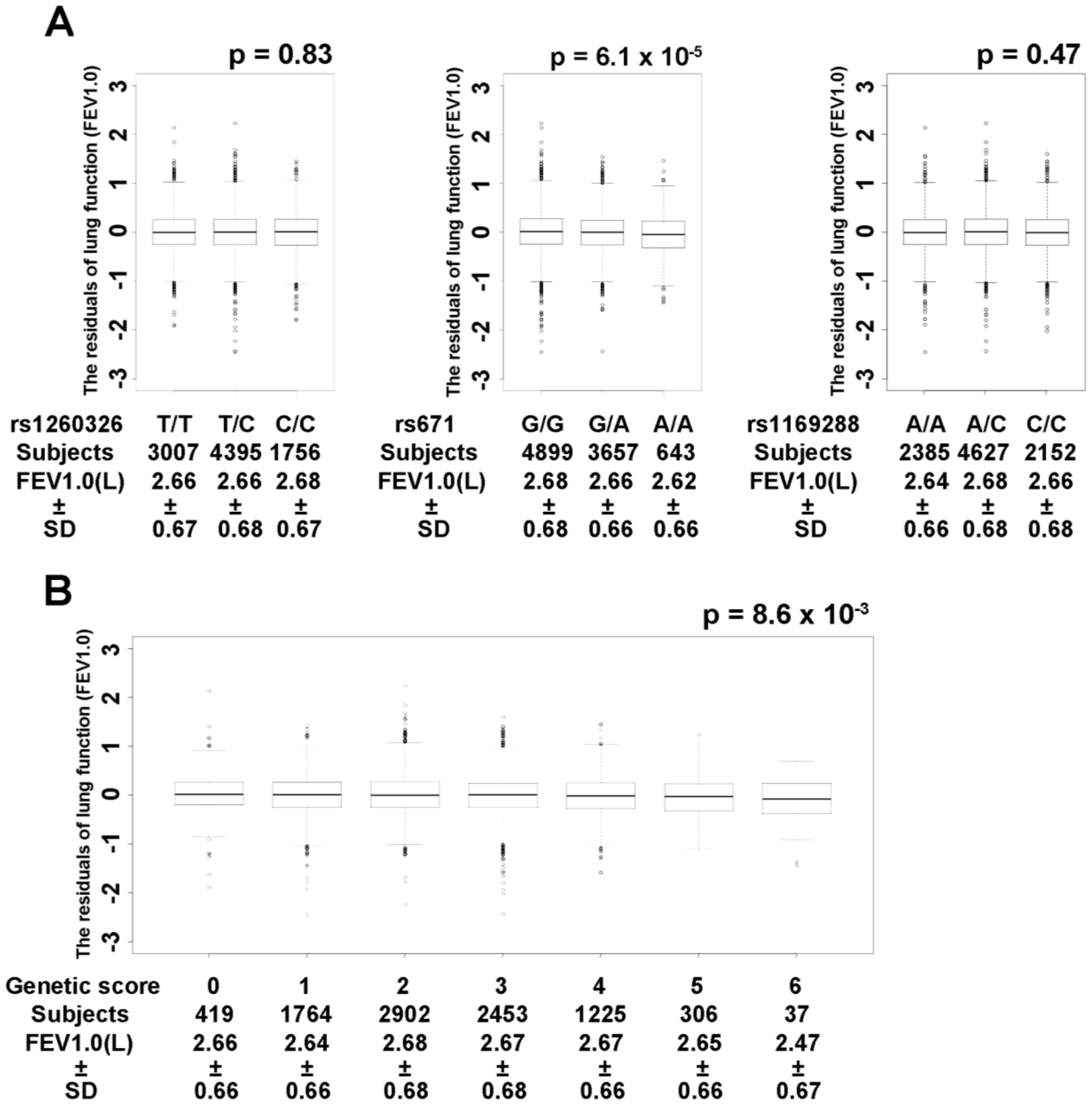

Supplementary Figure 11 | Associations between lung function and the three variants after conditioning on serum AAT levels

The associations between AAT and the three SNPs(A) or the number of risk alleles of the three SNPs(B) after conditioning on AAT levels are indicated.

P: p-value calculated by generalized linear regression model.

The upper and lower boundaries of the boxes represent the third and first quartiles,

respectively. The horizontal lines represent the medians. The whiskers represent more or less than 1.5 times of the interquartile range.

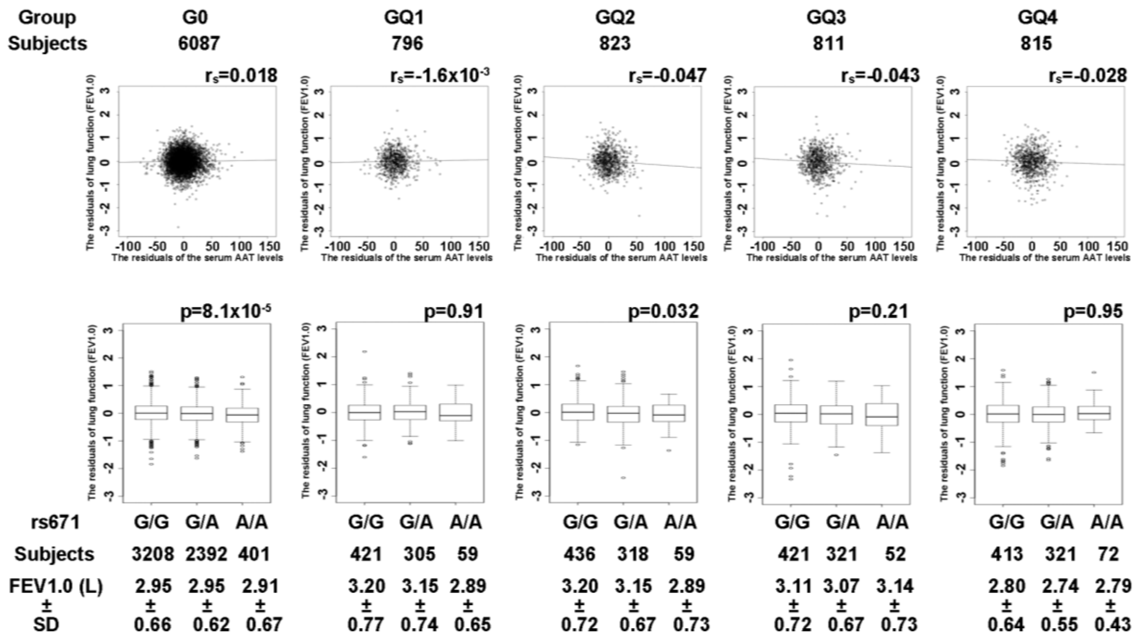

Supplementary Figure 12 | An association between rs671 and FEV1.0 according to smoking status

The association between rs671 and FEV1.0 is shown based on the subgroups according to smoking status. G0: non-smokers, GQ1-4: smokers quartile according to Brinkman Index.

P: p-value calculated by generalized linear regression model. r_S: Spearman's rank sum coefficient.

The upper and lower boundaries of the boxes represent the third and first quartiles, respectively. The horizontal lines represent the medians. The whiskers represent more or less than 1.5 times of the interquartile range.

Supplementary Table 1 | The SNPs showing p-values less than 1.0x10^{-6} in the GWAS

SNP	CHR	Position	Gene	Ref/Var	Location	VAF	RSQ	HWE-p	Effect (SE)	P
rs1260326	2	27730940	GCKR	T/C	nonsyn	0.425	0.997	1.90 x 10^{-3}	2.15 (0.43)	7.07 x 10^{-7}
rs7316287	12	111321512	CCDC63	T/C	intronic	0.220	0.925	0.777	2.62 (0.53)	8.12 x 10^{-7}
rs76024719	12	111321724	CCDC63	G/A	intronic	0.220	0.92	0.707	2.64 (0.53)	7.60 x 10^{-7}
rs202051532	12	111322994	CCDC63	AAGAG/A	intronic	0.218	0.895	0.671	2.63 (0.53)	8.53 x 10^{-7}
rs7311323	12	111323939	CCDC63	G/A	intronic	0.781	0.883	0.328	-2.69 (0.54)	5.43 x 10^{-7}
rs10849914	12	111325437	CCDC63	T/A	intronic	0.240	0.859	0.540	2.55 (0.52)	8.09 x 10^{-7}
rs192395428	12	111328450	CCDC63	C/T	intronic	0.237	0.909	0.458	2.58 (0.52)	7.30 x 10^{-7}
rs11065749	12	111329227	CCDC63	G/A	intronic	0.237	0.916	0.444	2.57 (0.52)	7.60 x 10^{-7}
rs139183566	12	111330204	CCDC63	A/AC	intronic	0.237	0.914	0.444	2.57 (0.52)	7.60 x 10^{-7}
rs11065750	12	111331016	CCDC63	G/A	intronic	0.237	0.936	0.444	2.57 (0.52)	7.60 x 10^{-7}
rs11065751	12	111331156	CCDC63	T/A	intronic	0.237	0.937	0.444	2.57 (0.52)	7.60 x 10^{-7}
rs11065752	12	111331165	CCDC63	G/A	intronic	0.237	0.938	0.490	2.57 (0.52)	7.78 x 10^{-7}
rs149021839	12	111333947	CCDC63	AATTT/A	intronic	0.217	0.789	0.211	2.74 (0.54)	3.79 x 10^{-7}
rs75295329	12	111344621	CCDC63	G/T	intronic	0.206	0.697	0.082	2.81 (0.55)	3.45 x 10^{-7}
rs12227162	12	111367244	MYL2/LOC1000131138	C/T	intergenic	0.208	0.641	0.015	3.29 (0.55)	2.64 x 10^{-9}
rs145558108	12	111377776	LOC100131138/CUX2	C/CT	intergenic	0.265	0.579	1.37 x 10^{-5}	2.59 (0.52)	5.89 x 10^{-7}
rs2188380	12	111386127	LOC100131138/CUX2	T/C	intergenic	0.207	0.702	0.032	3.21 (0.55)	6.05 x 10^{-9}
rs149607519	12	111389437	LOC100131138/CUX2	C/G	intergenic	0.205	0.675	0.050	3.20 (0.55)	7.36 x 10^{-9}
rs148177611	12	111390454	LOC100131138/CUX2	TAGAA/T	intergenic	0.205	0.681	0.055	3.22 (0.55)	5.92 x 10^{-9}
rs3809297	12	111609727	CUX2	G/T	intronic	0.261	0.546	0.047	3.28 (0.51)	1.13 x 10^{-10}
rs2339717	12	111696528	CUX2	T/C	intronic	0.626	0.976	0.600	-2.67 (0.45)	4.94 x 10^{-9}
rs6490029	12	111698457	CUX2	G/A	intronic	0.626	0.993	0.581	-2.66 (0.45)	5.57 x 10^{-9}
rs916682	12	111699146	CUX2	A/G	intronic	0.626	0.997	0.531	-2.65 (0.46)	6.13 x 10^{-9}
rs3858704	12	111705893	CUX2	A/G	intronic	0.654	0.987	0.908	-2.75 (0.46)	2.71 x 10^{-9}
rs4766566	12	111706877	CUX2	C/T	intronic	0.654	0.996	0.908	-2.75 (0.46)	2.71 x 10^{-9}
rs79105258	12	111718231	CUX2	C/A	intronic	0.254	0.721	0.770	2.96 (0.50)	4.72 x 10^{-9}
rs3782886	12	112110489	BRAP	T/C	syn	0.286	0.995	0.767	3.02 (0.48)	4.98 x 10^{-10}
rs11066001	12	112119171	BRAP	T/C	intronic	0.286	0.864	0.767	3.02 (0.48)	4.98 x 10^{-10}
rs11066008	12	112140669	ACAD10	A/G	intronic	0.288	0.523	0.769	2.96 (0.48)	8.96 x 10^{-10}
rs11066015	12	112168009	ACAD10	G/A	intronic	0.270	0.632	0.814	3.27 (0.49)	4.17 x 10^{-11}
rs4646776	12	112230019	ALDH2	G/C	intronic	0.269	0.841	0.783	3.31 (0.49)	2.49 x 10^{-11}
rs671	12	112241766	ALDH2	G/A	nonsyn	0.270	0.997	0.814	3.28 (0.49)	3.38 x 10^{-11}
rs11068574	12	118053982	KSR2	A/G	intronic	0.651	0.995	0.423	-2.28 (0.46)	9.77 x 10^{-7}
rs1169289	12	121416622	HNF1A	C/G	syn	0.454	0.898	0.705	2.16 (0.44)	9.60 x 10^{-7}
rs1169288	12	121416650	HNF1A	A/C	nonsyn	0.490	0.703	0.077	2.40 (0.45)	8.45 x 10^{-8}
rs2244608	12	121416988	HNF1A	A/G	intronic	0.471	0.872	0.636	2.30 (0.44)	1.74 x 10^{-7}
rs63470963	12	121417536	HNF1A	G/GACTC	intronic	0.465	0.856	0.424	2.19 (0.44)	6.02 x 10^{-7}
rs1169284	12	121419926	HNF1A	T/C	intronic	0.472	0.939	0.366	2.32 (0.44)	1.24 x 10^{-7}
rs7979473	12	121420260	HNF1A	A/G	intronic	0.527	0.932	0.358	-2.31 (0.44)	1.31 x 10^{-7}
rs7979478	12	121420263	HNF1A	A/G	intronic	0.527	0.943	0.358	-2.31 (0.44)	1.31 x 10^{-7}
rs1183910	12	121420807	HNF1A	G/A	intronic	0.472	0.954	0.404	2.30 (0.44)	1.59 x 10^{-7}
rs11065384	12	121423285	HNF1A	T/C	intronic	0.528	0.956	0.509	-2.29 (0.44)	1.81 x 10^{-7}
rs7970695	12	121423376	HNF1A	G/A	intronic	0.527	0.968	0.501	-2.30 (0.44)	1.58 x 10^{-7}
rs11065385	12	121423386	HNF1A	A/G	intronic	0.528	0.960	0.509	-2.29 (0.44)	1.81 x 10^{-7}
rs9738226	12	121423659	HNF1A	A/G	intronic	0.521	0.949	0.442	-2.22 (0.44)	4.35 x 10^{-7}
rs2393791	12	121423956	HNF1A	C/T	intronic	0.528	0.981	0.384	-2.28 (0.44)	2.06 x 10^{-7}

rs2393776	12	121424406	HNF1A	G/A	intronic	0.528	0.985	0.384	-2.28 (0.44)	2.06×10^{-7}
rs2243458	12	121424490	HNF1A	C/T	intronic	0.472	0.985	0.384	2.28 (0.44)	2.06×10^{-7}
rs2393775	12	121424574	HNF1A	G/A	intronic	0.528	0.994	0.384	-2.28 (0.44)	2.06×10^{-7}
rs7310409	12	121424861	HNF1A	A/G	intronic	0.528	0.999	0.384	-2.28 (0.44)	2.06×10^{-7}
rs1169292	12	121426478	HNF1A	C/T	intronic	0.478	0.949	0.466	2.26 (0.44)	2.51×10^{-7}
rs1169294	12	121426594	HNF1A	G/A	intronic	0.479	0.946	0.364	2.28 (0.44)	1.90×10^{-7}

CHR: chromosome, Ref/Var: Reference allele/Variant allele, VAF: Variant allele frequency, HWE: Hardy–Weinberg equilibrium, SE: standard error, syn/nonsyn: synonymous/nonsynonymous. RSQ indicates a quality of imputation. P: p-value calculated by generalized linear regression model.

The applied reference panel: NCBI build 37.

Supplementary Table 2 | Variances of AAT levels explained by the three SNPs

SNP	Variance explained by SNPs (%)
rs1260326	0.59
rs671	0.90
rs11068574	0.46
Total	1.95

Supplementary Table 3 | Functional annotation for the associated SNPs and those in high LD (r^2>0.8)

SNP	CHR	Position	Gene	Location	r^2		Ref /Var	VAF		P (GWAS)	Promoter		Enhancer			DNase	Proteins bound by ChIP	
					Nagahama (Index SNP)	European		NAG	EUR		Active promoter	Weak promoter	Strong enhancer	Weak enhancer	others		Cell_type	Protein
rs1260326	2	27730940	GCKR	Nonsyn	-	-	T / C	0.43	0.59	7.1 x 10^{-7}					LIV.A (Roadmap, TxEnhG1)	HepG2		
rs6547692	2	27734972	GCKR	Intronic	0.928 (rs1260326)	0.80	G / A	0.43	0.55	2.3 x 10^{-6}								
rs780096	2	27741072	GCKR	Intronic	0.914 (rs1260326)	0.81	C / G	0.43	0.56	1.6 x 10^{-6}			HepG2				HepG2	RXRA
rs780095	2	27741105	GCKR	Intronic	0.914 (rs1260326)	0.81	A / G	0.43	0.56	1.6 x 10^{-6}			HepG2	K562			HepG2	FOXA2 , RXRA
rs780094	2	27741237	GCKR	Intronic	0.914 (rs1260326)	0.91	T / C	0.43	0.59	1.6 x 10^{-6}			HepG2	K562			HepG2	FOXA2, MAFK, RXRA
rs780093	2	27742603	GCKR	Intronic	0.903 (rs1260326)	0.91	T / C	0.43	0.59	2.5 x 10^{-6}					LIV.A (Roadmap, TxEnhG1)	GM1289, CD4_Th0_A		
rs1313566	2	27748904	GCKR/ C2orf16	intergenic	0.901 (rs1260326)	0.81	G / A	0.43	0.56	2.2 x 10^{-6}								
rs1260334	2	27748597	GCKR/ C2orf16	intergenic	0.900 (rs1260326)	0.72	C / A	0.43	0.57	2.9 x 10^{-6}								
rs1260333	2	27748624	GCKR/ C2orf16	intergenic	0.898 (rs1260326)	0.80	A / G	0.43	0.55	1.8 x 10^{-6}								
rs199682409	2	27750543	GCKR/ C2orf16	intergenic	0.892 (rs1260326)	0.55	A / AAG	0.42	0.50	1.8 x 10^{-6}				K562				
rs200747666	2	27750544	GCKR/ C2orf16	intergenic	0.891 (rs1260326)	0.55	A / AG	0.42	0.50	1.6 x 10^{-6}				K562				
rs2950835	2	27750545	GCKR/ C2orf16	intergenic	0.891 (rs1260326)	0.55	A / G	0.42	0.50	1.6 x 10^{-6}				K562				
rs11127048	2	27752463	GCKR/ C2orf16	intergenic	0.891 (rs1260326)	0.72	G / A	0.42	0.57	1.1 x 10^{-6}								
rs2911711	2	27750546	GCKR/ C2orf16	intergenic	0.890 (rs1260326)	0.55	T / A	0.42	0.50	2.1 x 10^{-6}				K562				
rs6753534	2	27752871	GCKR/ C2orf16	intergenic	0.831 (rs1260326)	0.79	C / T	0.42	0.56	3.4 x 10^{-6}								

SNP	CHR	Position	Gene	Location	r^2		Ref /Var	VAF		P (GWAS)	Promoter		Enhancer			DNase	Proteins bound by ChIP	
					Nagahama (Index SNP)	European		NAG	EUR		Active promoter	Weak promoter	Strong enhancer	Weak enhancer	others		Cell_type	Protein
rs671	12	112241766	ALDH2	nonsyn	-	-	G / A	0.27	0	3.4×10^{-11}						Chorion, GM19239, LNCaP (AT), CD4_Th0_A		
rs4646776	12	112230019	ALDH2	intronic	0.998 (rs671)	-	G / C	0.27	0	2.5×10^{-11}			K562		LIV.A (Roadmap, TxEnhG1), IMR90 (Roadmap, TxEnhG1)	HepG2, Caco-2		
rs11066015	12	112168009	ACAD10	intronic	0.995 (rs671)	-	G / A	0.27	0	4.2×10^{-11}						GM19239		
rs3782886	12	112110489	BRAP	syn	0.916 (rs671)	-	T / C	0.29	0	5.0×10^{-10}								
rs11066001	12	112119171	BRAP	intronic	0.916 (rs671)	-	T / C	0.29	0.0026	5.0×10^{-10}								
rs11066008	12	112140669	ACAD10	intronic	0.911 (rs671)	-	A / G	0.29	0	9.0×10^{-10}								
rs1169288	12	121416650	HNF1A	nonsyn	-	-	A / C	0.49	0.33	8.5×10^{-8}	HepG2, LIV.A (Roadmap)					LNCaP, iPS	HepG2	POL2, TAF1
rs1169289	12	121416622	HNF1A	syn	0.846 (rs1169288)	0.57	C / G,T	0.45	0.46	9.6×10^{-7}	HepG2, LIV.A (Roadmap)					LNCaP, iPS	HepG2	POL2, TAF1
rs2244608	12	121416988	HNF1A	intronic	0.804 (rs1169288)	0.95	A / G	0.47	0.34	1.7×10^{-7}	HepG2, LIV.A (Roadmap)							

CHR: chromosome, syn/nonsyn: synonymous/nonsynonymous. Ref/Var: Reference allele/Variant allele, VAF: Variant allele frequency, NAG: Nagahama population, EUR: European, P: p-value calculated by generalized linear regression model. LIV.A: Adult Liver, HepG2: Cell line (Hepatocellular carcinoma), K562: Cell line (Chronic myelogenic leukemia), GM1289: B-lymphocyte lymphoblastoid, CD4_Th0_A: CD4+ cells isolated from human blood and enriched for Th0 populations (Adult), IMR90: Cell line (Fetal lung fibroblast),

Caco2: Cell line (Colorectal adenocarcinoma), Chorion: Chorion cells (Outermost of two fetal membranes), GM19239: B-lymphocyte lymphoblastoid, LNCaP: Cell line (Prostate adenocarcinoma), AT: Androgen treated, iPS: Induced pluripotent stem cell derived from skin fibroblast, TxEnhG1: Transcription Enhancer-like. The functional annotations shown above are results of ENCODE project. When functional annotations in liver or lung-derived cells were found in Roadmap project, these are also indicated.

The applied reference panel: NCBI build 37.

Supplementary Table 4 | Amino acid conservation for HNF1A and GCKR

Rs1169288

Species	Amino acid sequence																				
Human	L	E	S	G	L	S	K	E	A	L	**I**	Q	A	L	G	E	P	G	P	Y	L
Rhesus	L	E	S	G	L	S	K	E	A	L	**I**	Q	A	L	G	E	P	E	P	Y	L
Mouse	L	E	S	G	L	S	K	E	A	L	**I**	Q	A	L	G	E	P	G	P	Y	L
Dog	L	H	S	G	L	S	K	E	A	L	**I**	Q	A	L	G	E	P	G	P	Y	L
Elephant	L	E	S	G	L	T	K	E	A	L	**I**	Q	A	L	G	E	P	G	P	Y	L
Opossum	L	E	S	G	L	T	K	E	T	L	**I**	R	A	L	G	E	T	R	P	Y	D
Chicken	L	E	S	G	L	T	K	E	T	L	**I**	K	A	L	S	E	A	E	P	Y	V
Zebrafish	L	D	S	G	V	T	K	D	V	L	**L**	Q	A	L	E	D	L	D	P	S	P

Rs1260326

Species	Amino acid sequence																				
Human	L	A	H	S	T	V	G	Q	T	L	**L**	I	P	L	K	K	L	F	P	S	I
Rhesus	L	A	H	S	T	V	G	Q	N	L	**P**	I	P	L	K	K	L	F	P	S	V
Mouse	L	V	H	S	T	V	G	Q	S	L	**P**	A	P	L	K	K	L	F	P	S	L
Dog	L	A	H	S	T	V	G	Q	S	L	**P**	T	P	L	K	K	L	F	P	S	I
Elephant	L	A	H	S	T	V	G	Q	S	L	**P**	T	L	L	K	K	L	F	P	S	I
Opossum	L	A	H	S	T	V	G	Q	Y	L	**P**	S	P	L	K	K	L	F	P	S	I
Chicken	-	-	-	-	-	-	-	-	-	-	-	-	-	-	-	-	-	-	-	-	-
Zebrafish	-	-	-	-	-	-	-	-	-	-	-	-	-	-	-	-	-	-	-	-	-

Data source: UCSC Genome Browser on Human Feb. 2009 (GRCh37/hg19) Assembly

-: No homologous sequences

Supplementary Table 5 | Interactions between rs671 and covariates on serum AAT levels

Interaction	Effect (SE)	P
rs671 x Sex	-1.53 (0.60)	0.011
rs671 x Age	-0.019 (0.021)	0.38
rs671 x BMI	-0.058 (0.085)	0.50
rs671 x log (hs-CRP)	0.22 (0.54)	0.68
rs671 x Alcohol intake	1.59 (0.43)	2.2×10^{-4}
rs671 x Brinkman index	2.9×10^{-3} (8.7×10^{-4})	6.8×10^{-4}

P: p-value calculated by generalized linear regression model.

Supplementary Table 6 | The lack of interactions between the two genetic variants and alcohol consumption or smoking on serum AAT levels

Interaction	Effect (SE)	P
rs1260326 x Alcohol intake	0.21 (0.30)	0.48
rs1169288 x Alcohol intake	-0.87 (0.30)	0.77
rs1260326 x Brinkman index	-3.8×10^{-3} (7.8×10^{-4})	0.62
rs1169288 x Brinkman index	2.6×10^{-4} (7.8×10^{-4})	0.74

P: p-value calculated by generalized linear regression model.

Supplementary Table 7 | The number of subjects according to GWAS arrays

**Platform**	**Sample number**
Hap610K	1,718
2.5M-4	1,024
2.5M-8	186
Hap610K + 2.5M-4 + CoreExome + 2.5M-s	112
2.5M- 4 + 2.5M-s + Exome	480
2.5M-8 + 2.5M-s + Exome	192

Hap610K: human hap610K quad array, 2.5M-4: human omni 2.5M -4 array, 2.5M-8: human omni 2.5M -8 array, 2.5M-s: human omni 2.5s array, Exome: human exome, CoreExome: human core exome (Illumina, San Diego, CA, USA).

Supplementary Note

Selection of the samples in the current study

Samples for genome-scanning in the Nagahama Study were selected from the DNA samples of the participants recruited in 2008 or 2009. Since high quality of DNA samples is an essential factor to obtain excellent GWAS results especially in terms of success rate, we put strict quality control for sample selection of GWAS. As a result, we excluded 2,405 subjects from samples that showed any signs of fragmented DNA. We performed genome-scanning using the remaining subjects (Supplementary Fig. 1 and Supplementary Table 7). The 4,023 DNA samples from the participants recruited in 2010 were subjected for replication study 1. Since we used Taqman assay in the replication studies which does not always require excellent quality of DNA, the 2,405 subjects that were not used for GWAS were subjected for replication study 2. Therefore, 2,402 subjects out of the 2,405 whose smoking information were available were genotyped for replication 2 (Supplementary Fig. 1). We used a total of 9,359 subjects for the current study to maximize the power to detect significant variations.
